# Butchering activity is the main risk factor for hepatitis E virus (*Paslahepevirus balayani*) infection in southwestern Nigeria: a prospective cohort study

**DOI:** 10.3389/fmicb.2023.1247467

**Published:** 2023-09-26

**Authors:** Adeolu S. Oluremi, Maria Casares-Jimenez, Oluyinka O. Opaleye, Javier Caballero-Gomez, David Olusoga Ogbolu, Pedro Lopez-Lopez, Diana Corona-Mata, Antonio Rivero-Juarez, Antonio Rivero

**Affiliations:** ^1^Department of Medical Microbiology and Parasitology, Ladoke Akintola University of Technology, Ogbomoso, Nigeria; ^2^Unit of Infectious Diseases, Hospital Universitario Reina Sofia, Instituto Maimonides de Investigación Biomédica de Córdoba (IMIBIC), Universidad de Córdoba (UCO), Córdoba, Spain; ^3^CIBERINFEC, ISCIII – CIBER de Enfermedades Infecciosas, Instituto de Salud Carlos III, Madrid, Spain; ^4^Departamento de Sanidad Animal, Grupo de Investigación en Sanidad Animal y Zoonosis (GISAZ), UIC Zoonosis y Enfermedades Emergentes ENZOEM, Universidad de Córdoba, Córdoba, Spain; ^5^Department of Medical Laboratory Science, Osun State University, Ogbomoso, Nigeria

**Keywords:** HEV, IgG, IgM, ELISA, butchering, animal contact, zoonoses

## Abstract

**Introduction:**

*Paslahepevirus balayani* (Hepatitis E virus; HEV) is an emerging virus that poses as a public health threat. The virus is now reported to be the leading cause of acute viral hepatitis, with a unique impact on African settings. Our aim was to evaluate the prevalence and risk factors for HEV infection in three cohorts (animal handlers, villagers, and students).

**Methods:**

A prospective cross-sectional study was carried out on a total of 752 subjects from southwestern Nigeria. In all individuals, anti-HEV IgG and anti-HEV IgM antibodies were evaluated by using ELISA (confirming positive results via immunoblotting), and serum viral RNA was evaluated by using two RT-PCR assays.

**Results:**

The overall seroprevalence of HEV IgG and HEV IgM was 14.9% (95% CI: 12.5–17.6%) and 1.3% (95% CI: 0.7–2.5%), respectively. We observed the highest seroprevalence among animal contact individuals, with butchers being the population with the highest HEV IgG seroprevalence (31.1%). Similarly, HEV IgM was higher in the animal contact group (2.2%) than in the non-animal contact cohort (0%).

**Discussions:**

Viral RNA was not detected in any of the samples. Butchering was significantly associated with higher HEV prevalence. Although all efforts to prevent HEV in Africa have focused on the chlorination of water, our study suggests that most new infections could currently be linked to animal manipulation. Therefore, education and guidelines must be provided in southwest Nigeria to ensure that animal handling and processing methods are safe.

## Graphical abstract

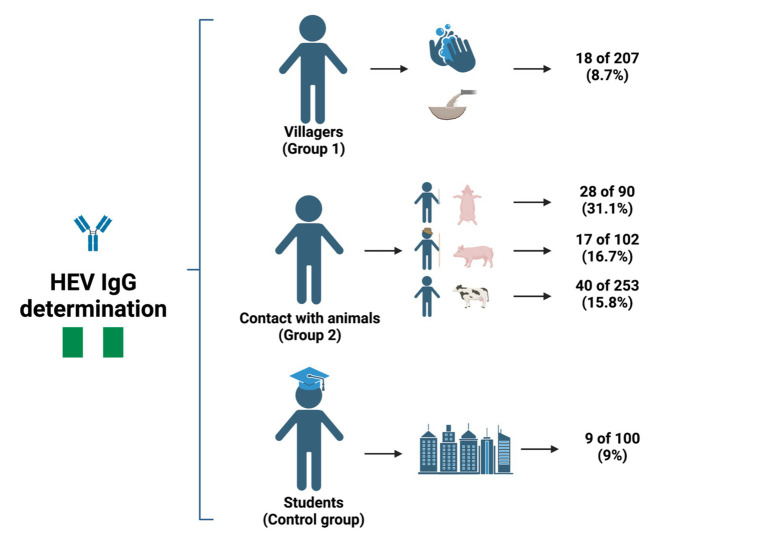

## Background

*Paslahepevirus balayani*, which was previously known as the hepatitis E virus (HEV), has been linked not only to sporadic cases but also outbreaks of acute hepatitis in humans (Hoofnagle et al., 2012). Although this virus is distributed worldwide, high endemic circulation has been observed in different countries in Asia and Africa (Saade et al., 2022). Moreover, this virus has been assigned 8 different genotypes (Smith et al., 2020). Human cases are associated with genotypes HEV-1 and HEV-2 which are transmitted via the fecal-oral route through the consumption of contaminated water, whereas HEV-3 and HEV-4 are zoonotic with a broader host range and transmitted through the consumption of animal products or contact with infected animals (Khuroo et al., 2016; Antia et al., 2018; Nimgaonkar et al., 2018; Desai et al., 2022). Human HEV infection is usually self-limiting and frequently asymptomatic; symptoms include diarrhea, epigastric pain, nausea, vomiting, hepatomegaly and splenomegaly. The icteric stage begins with jaundice, dark urine, and clay-colored stools (Hoofnagle et al., 2012). Furthermore, extrahepatic manifestations are associated with HEV-3 infections, thus highlighting both central and peripheral nervous system acute injury, among others (Pischke et al., 2017).

This virus is one of the emerging pathogens of national health importance (Webb and Dalton, 2019). Globally, approximately 20 million people are infected annually, of whom approximately 3.3 million cases and 44,000 deaths are reported (World Health Organization, 2017). In West Africa, HEV-1, HEV-2, and HEV-3 have been commonly reported during the last few years (Bagulo et al., 2020). While genotypes 1 and 2 are responsible for large scale outbreaks in humans (Modiyinji et al., 2021a), genotypes 3 and 4 cause sporadic cases (Okagbue et al., 2019). The fecal-oral route and contaminated water sources have also been linked to most outbreaks in endemic areas (Hakim et al., 2017). In addition, outbreak and autochthonous cases have been linked to the eating of parboiled liver and local pigs’ meat, deer, and wild boar (EFSA, 2017; Desai et al., 2022).

HEV infection has maintained its endemic status in Nigeria, resulting in notable outbreaks in the recent past (Ifeorah et al., 2017). The most recent outbreak occurred in July 2017 in Northeast Nigeria, involving a total of 146 confirmed or suspected cases (World Health Organization, 2017; Okagbue et al., 2019). The outbreak in July 2017 was a consequence of the humanitarian crisis in Northeast Nigeria, primarily driven by insurgency and population movements within the Lake Chad basin. This turmoil led to a shift in population toward southern Nigeria, exacerbating the situation and emphasizing the ongoing public health threat posed by HEV in the region. Given these circumstances, it becomes crucial to gather updated information about the epidemiology of HEV in southwestern Nigeria. Accurate diagnosis, identification, and genotyping of the virus hold immense importance for effective disease management, control, and prevention strategies. Furthermore, there is a necessity to refresh data on the epidemiology and risk factors related to HEV among various cohorts within southwestern Nigeria. In light of these considerations, the objectives of our study were to conduct a thorough analysis of the sero-epidemiology of HEV and to identify the associated risk factors within different populations residing in southwestern Nigeria. By addressing these aspects, we aim to contribute to a better understanding of the prevalence and transmission dynamics of HEV in the region, ultimately guiding more targeted and effective public health interventions.

## Materials and methods

### Populations

This cross-sectional study comprises individuals from 3 cohorts established in Nigeria: villagers, individuals in contact with animals, and students attending Babcock University. Villagers were from two villages: (i) Ogbomosho (latitude: 8.12275; longitude: 4.243589) and (ii) Prince Village, Iwo (latitude: 7.6353; longitude: 4.1816). Contact with animal groups was divided into three subgroups: (i) animal handlers (which were defined as nonprofessional farmers contacting animals other than swine), (ii) butchers (mostly cow and sheep butchers), and (iii) pig handlers. These populations were selected to evaluate the prevalence and risk factors due to different HEV genotypes. Specifically, villagers were selected because of poor sanitation conditions, which may make them have higher risks of HEV infection by genotypes 1 and 2, and animal handlers were enrolled because they have the highest risk of zoonotic genotype infection. Furthermore, students served as a control group.

### Demography and blood collection

Demographic information, including gender, age, and sex, among other information was collected via questionnaire. The samples were collected between January 2020 and February 2021. Blood samples were collected from each participant via venipuncture, centrifuged to obtain serum, immediately transported on ice to the laboratory, and maintained at −80°C.

### Serological survey

Serum samples were screened for anti-HEV IgG and anti-HEV IgM antibodies using ELISA for HEV genotypes 1–4 (recomWell HEV IgG/IgM®; Mikrogen Diagnostik, Neuried, Germany) following manufacturer procedures (Automatic ELISA workstation DS2^®^, Dynex Technologies). A cut-off value >24 U/mL was considered positive while those with those range between 20 and 24 U/mL were considered borderline. According to the manufacturer’s instructions, confirmation testing was performed by using immunoblotting (recombine HEV IgG/IgM^®^; Mikrogen Diagnostik, Neuried, Germany).

### Molecular survey

RNA was extracted from 400 μL pools of serum comprising sera from four different individuals (100 μL of each sample) by using the QIAamp MinElute virus spin kit and the QIAcube system (QIAGEN, Hilden, Germany). A total volume of 50 μL purified RNA was eluted following the manufacturer’s instruction. To detect HEV RNA (genotypes 1–8 of the genus *Paslahepevirus balayani*), real-time RT-PCR (CFX Connect Real-Time PCR System) was performed using 25 μL of RNA template and the QIAGEN One-Step RT–PCR kit (Frías et al., 2021). The sensitivity of PCR in 4 sample pools was set at 350 IU/mL, and that for individual samples was set at 21 IU/mL. The positive control used was the first WHO International Standard for HEV RNA Nucleic Acid Amplification Techniques (NAT)-Based Assays, consistent with HEV genotype 3a (PEI code 6219/10) provided by the Paul-Ehrlich-Institut (PEI code 6219/10) was used.

### Statistical analysis

Two outcome variables that were established include (i) positivity for HEV IgG antibodies and (ii) positivity for HEV IgM antibodies. The prevalence of IgG and IgM was calculated. Categorical variables were analyzed using the Chi-square (X^2^) test or Fisher’s exact test expressed in percentage. *P* ≤ 0.05 was considered significant. Bivariate analysis was used to compare the prevalence and incidence rate between groups to identify variables related to the different outcome variables. Moreover, stepwise logistic regression was employed for the multivariate analysis of the variables. An odds ratio (95% CI) was provided. All the analyses were performed by using statistical software package version 18.0 (IBM Corporation, Somers, NY, USA) and GraphPad Prism version 7 (Mac OS X version; GraphPad Software; San Diego, California, USA).

## Results

### Population

The study population comprised 752 individuals, including 445 (59.2%) individuals with animal contact, 207 (27.5%) villagers, and 100 students. Among the animal contact group, 253 (56.9%) individuals were classified as animal handlers, 102 (22.9%) individuals were classified as pig handlers, and 90 (20.2%) were butchers. The demographic details of the participants are summarized in Table 1.

**Table 1:** Demographic characteristics of the population.

Variable	Global	Contact with animals				Villagers	Students
		Global	AH	PH	BU		
N	752	445	253	102	90	207	100
Gender, n (%)							
Male	390 (51.9)	236 (53)	84 (33.2)	95 (93.1)	57 (63.3)	107 (51.7)	47 (47.0)
Female	362 (48.1)	209 (47)	169 (66.8)	7 (6.9)	33 (36.7)	100 (48.3)	53 (53.0)
Age (years), n (%)							
>50	228 (30.3)	168 (37.8)	74 (29.2)	52 (51.0)	42 (46.7)	60 (29.0)	0
41–50	109 (14.5)	76 (17.1)	43 (17.0)	22 (21.6)	11 (12.2)	33 (15.9)	0
31–40	145 (19.3)	100 (22.5)	70 (27.7)	13 (12.7)	17 (18.9)	45 (21.7)	0
21–30	176 (23.4)	68 (15.3)	39 (15.4)	14 (13.7)	15 (16.7)	52 (25.1)	56 (56)
<20	94 (12.5)	33 (7.4)	27 (10.7)	1 (1.0)	5 (5.6)	17 (8.2)	44 (44)

Animal handler (AH); Pig handler (PH); butcher (BU); number of individuals (N or n); percentage (%).

### Serological and molecular survey for HEV

A total of 112 individuals were positive for IgG antibodies, giving an overall seroprevalence of 14.9% (95% CI: 12.5–17.6%). Ten subjects demonstrated IgM antibodies, giving an IgM seroprevalence of 1.3% (95% CI: 0.7–2.5%). Three IgM positive individuals were also positive for IgG antibodies. The prevalence of IgG and IgM antibodies according to the study population is shown in Table 2. No HEV-RNA was amplified in any of the cohorts positive for ELISA.

**Table 2:** Seroprevalence of hepatitis E virus IgG and IgM antibodies in the whole population and subgroups.

HEV serology	Global	Contact with animals				Villagers	Students
		Global	AH	PH	BU		
N	752	445	253	102	90	207	100
HEV IgG antibodies, n (%)							
Negative	640 (85.1)	360 (80.9)	213 (84.2)	85 (83.3)	62 (68.9)	189 (91.3)	91 (91.0)
Positive	112 (14.9)	85 (19.1)	40 (15.8)	17 (16.7)	28 (31.1)	18 (8.7)	9 (9.0)
HEV IgM antibodies, n (%)							
Negative	742 (98.7)	435 (97.8)	248 (98)	98 (96.1)	89 (98.9)	207	100
Positive	10 (1.3)	10 (2.2)	5 (2.0)	4 (3.9)	1 (1.1)	0	0

Animal handler (AH); Pig handler (PH); butcher (BU); number of individuals (N or n); percentage (%).

### Risk factors associated with HEV seroprevalence

The highest IgG seroprevalence that was observed in the study was in those individuals included in the contact with animal group (19.1%), followed by the villagers (8.7%) and students (9.0%) (*p* < 0.001). Furthermore, the IgM antibody detection rate was significantly higher in this group than in the other groups (2.2% vs. 0%, respectively; *p* = 0.03). No differences were found between villagers and the student control group. Similarly, the IgG seroprevalence was similar between the age groups (> 50 years = 15.8%; 41–50 years = 12.8%; 31–40 years = 19.3%; 21–30 years = 13.1%; <20 years = 11.7%; *p* = 0.412) and gender (males = 14.6% vs. females = 15.2%; *p* = 0.838).

In the contact with the animal group, the seroprevalence of HEV IgG antibodies was higher in butchers (31.1%) than in animal and pig handlers (16.7 and 15.8% respectively; *p* = 0.005). Nevertheless, the IgM antibody was similar between the groups (*p* = 0.384). Furthermore, there were no significant differences in IgG antibody positivity between the two groups of villagers (*p* = 0.326).

The multivariate analysis incorporated several variables including gender (male and female), age categories (>50, 41–50, 31–40, 21–30, and < 20 years), and different risk category groups (student, villager, animal handler, pig handler, and butcher). The results revealed that the sole variable exhibiting independent association with HEV IgG positivity was the risk category group, specifically the “butcher” group (as depicted in Table 3).

**Table 3:** Multivariate analysis for hepatitis E virus IgG antibody prevalence.

Variable	OR	OR, 95% CI	*p* value
Gender			
Male	1		
Female	1.196	0.757–1.890	0.444
Age			
>50	1.000		
41–50	0.861	0.436–1.703	0.668
31–40	1.441	0.815–2.548	0.209
21–30	1.114	0.601–2.062	0.732
<20	1.064	0.474–2.391	0.880
Category			
Student	1.000		
Villager	0.961	0.385–2.400	0.933
Animal handler	1.810	0.768–4.269	0.175
Pig handler	2.316	0.846–6.346	0.102
Butcher	4.761	1.890–11.995	0.001

odds ratio (OR); 95% confidence interval (95% CI); significance value (*p*).

Furthermore, IgG prevalence were computed for each age group within every study cohort, and separately for each cohort in a univariate analysis, as well as collectively for the entire study population (as outlined in Table 4). Age was not found as associated risk factor with HEV IgG seroprevalence.

**Table 4:** Age-specific prevalences of IgG-HEV in each cohort included in the study and univariate analysis of age by each cohort and overall.

Age	Cohorts					Total n/N (%)	*p* value
	AH n/N (%)	PH n/N (%)	BU n/N (%)	Villagers n/N (%)	Students n/N (%)		
≤20	4/27 (14.8)	0/1 (0)	3/5 (60)	2/17 (11.8)	2/44 (4.5)	11/94 (11.7)	0.424
21–30	5/39 (12.8)	0/14 (0)	5/15 (33.3)	6/52 (11.5)	7/56 (12.5)	23/176 (13.1)	
31–40	18/70 (25.7)	2/13 (15.4)	4/17 (23.5)	4/45 (8.9)	0/0 (0)	28/145 (19.3)	
41–50	4/43 (9.3)	6/22 (27.3)	1/11 (9.1)	3/33 (9.1)	0/0 (0)	14/109 (12.8)	
>50	9/74 (12.1)	9/52 (17.3)	15/42 (35.7)	3/60 (5)	0/0 (0)	36/228 (15.8)	
*p* value	0.291	0.185	0.764	0.211	0.171	112/752 (14.8)	

Animal handler (AH); Pig handler (PH); butcher (BU); number of individuals positive (n); number of people included (N); percentage (%); significance value (*p*).

## Discussion

The overall prevalence of HEV IgG antibodies within our study population stood at 14.9%, revealing a substantial circulation of HEV in southwestern Nigeria. Of notable significance, the anticipated higher IgG seroprevalence among specific groups, such as villagers, was not observed when compared to the control group. Conversely, individuals in contact with animals exhibited significantly higher prevalence rates. Similarly, while older age groups were expected to demonstrate higher IgG seroprevalence (Feldt et al., 2013), our study did not confirm this relationship. These conflicting outcomes warrant further investigation due to their potential crucial epidemiological implications.

In contrast to European countries, HEV transmission in Africa has traditionally been associated with large outbreaks caused by contaminated water consumption and inadequate sanitation practices (Bagulo et al., 2018; Desai et al., 2022). Consequently, individuals in rural areas with deficient sanitation and limited access to safe drinking water are likely at the highest risk of HEV infection (Mbachu et al., 2021; Osundare et al., 2021). Among villagers, the prevalence of HEV IgG was 8.7%, which closely resembled the prevalence observed in students (9.0%). The absence of discernible differences in prevalence between the cohorts was unexpected given the poor living conditions and inadequate sewage disposal methods in most villages (Olayinka et al., 2020). This similarity might be linked to the lack of active HEV outbreaks in villages and the overall health education and disease prevention awareness promoted by local health authorities. Efforts have been intensified to address enteric virus prevention in Africa due to the associated high morbidity and mortality (Oluwasanya et al., 2022). Particularly, in relation to HEV, the World Health Organization (WHO) has formulated guidelines advocating home chlorination and water boiling, aiming to curb HEV outbreaks in Africa and mitigate virus transmission (World Health Organization, 2014). The low prevalence observed in our study might indirectly indicate the effectiveness of such measures. On the other hand, similar rate could be attributed to students’ travel history to developed countries where they might have consumed undercooked meat potentially harboring HEV. Thus, the risk practice for HEV acquisition could be different between both populations. In fact, the questionnaire include in our study primarily yielded information related to job status, which served as an indirect indicator of social status among the participants. Based on the data collected, it was observed that a significant portion of the participants held jobs associated with middle and low income levels, with the exception of students who were likely to belong to higher-earning families. The questionnaire did not directly inquire about social status; instead, it relied on participants’ job positions to infer their economic circumstances and, by extension, their social status. This indirect approach allowed us to make informed assessments of the social and economic diversity within the participant groups, but we cannot identify specific activities, culinary practice or routinary task which could be differ between groups.

Remarkably, our findings indicate that butchers face the highest risk of infection, with a prevalence exceeding 30% and encompassing most acute infections noted in our study. This heightened risk among butchers may stem from direct exposure to blood and other bodily fluids due to occupational accidents, as well as routine exposure to HEV (Pérez-Gracia et al., 2007; Montagnaro et al., 2015). Notably, individuals with significant contact with animal carcasses (butchers and slaughterhouse workers) tend to exhibit high IgG antibody prevalence, particularly in African settings (Adjei et al., 2009; Temmam et al., 2013; Oluremi et al., 2021). This might not be limited to pigs, since animals like cows, sheep, and goat have also been implicated as potential HEV reservoirs (Antia et al., 2018; Ouoba et al., 2019; Treagus et al., 2021; Ferri et al., 2022; Batmagnai et al., 2023). In fact, butchering related activities in southwestern Nigeria are more related with these species. Consequently, sheep and cows could have an important role on HEV transmission in this setting (Modiyinji et al., 2021b). On the other hand, poor sanitation in most African slaughterhouses could contribute to HEV transmission (Cook et al., 2017), while inadequate knowledge among those working with animal blood and unhygienic practices during meat procedures might play a pivotal role (Agu et al., 2021; Abunna et al., 2022). The absence of proper practices in abattoirs and slaughterhouses has been identified as a determining factor in the spread of infectious pathogens in Africa (Odetokun et al., 2022; Gerken et al., 2023), including HEV (Traoré et al., 2015). Our study emphasizes that slaughterhouse working conditions and practices pose a significant risk for HEV infection, underscoring a pressing public health concern in Nigeria. Effective preventive measures and educational programs should be established at this level, necessitating collaboration between food chain authorities and healthcare stakeholders to minimize worker risks and mitigate potential virus transmission (Murungi et al., 2021).

The relationship between age and HEV infection in Africa has been of interest due to its implications for disease epidemiology and public health interventions. While in some regions, age has been observed as a factor influencing HEV prevalence (Feldt et al., 2013), the relationship might not always follow the same pattern across different populations (Boon et al., 2018). Conventionally, older age has been linked to a higher prevalence of HEV antibodies, indicating previous exposure or infection. Studies have shown that older individuals are more likely to have IgG antibodies against HEV, suggesting a higher historical exposure to the virus. This trend is often attributed to cumulative exposures over time, possibly due to consumption of contaminated food or water or increased contact with animals. However, the age-prevalence relationship for HEV infection can vary across settings. Factors such as local transmission dynamics, variations in sanitation practices, animal reservoirs, and changing demographics can all influence the age distribution of HEV infection in different ways (Golkocheva-Markova et al., 2023). Thus, it’s important to consider the specific context of each study and population when interpreting the relationship between age and HEV infection. Therefore, a nuanced understanding of local dynamics is essential to formulate effective strategies for disease control and prevention.

## Conclusion

Our study found that butchers face a higher risk of HEV infection, indicating that coming into contact with the blood and other bodily fluids of infected animals is a primary risk factor for HEV transmission in Southwest Nigeria. Consequently, our study underscores the significance of implementing educational programs that emphasize safety practices in animal and carcass management to prevent HEV within this region.

## Data availability statement

The raw data supporting the conclusions of this article will be made available by the authors, without undue reservation.

## Ethics statement

The studies involving humans were approved by both Oyo State Hospital Management Board (AD13/479/585) and Babcock University Health Research Ethics Committee (BUHREC 529/21). The studies were conducted in accordance with the local legislation and institutional requirements. The participants provided their written informed consent to participate in this study.

## Author contributions

AR-J was involved in the study design, conception, interpretation of the data, drafting of the manuscript, study supervision, and funding obtention. ASO was involved in drafting the manuscript. ASO, MC-J, PL-L, DC-M, and JC-G were involved in serological and molecular determination. ASO, OOO, and DOO were involved in the data acquisition and critical review of the manuscript. AR was involved in the study design and conception, interpretation of the data, and funding obtention. All authors contributed to the article and approved the submitted version.

## Funding

This work was supported by Secretaría General de Investigación, Desarrollo e Innovación en Salud (PI-0287-2019) for grants for the financing of Investigación, Desarrollo e Innovación Biomédica y en Ciencias de la Salud en Andalucía; the Ministerio de Sanidad (RD12/0017/0012) integrated into the Plan Nacional de I+D+I and co-financed by the ISCIII-Subdirección General de Evaluación and the Fondo Europeo de Desarrollo Regional (FEDER); and the Fundación para la Investigación en Salud (FIS) del Instituto Carlos III (Research Project grant numbers: PI21/00793 and PI22/01098). AR-J was the recipient of a Miguel Servet Research Contract by the Ministerio de Ciencia, Promoción y Universidades of Spain (CP18/00111). JC-G was supported by the CIBER -Consorcio Centro de Investigación Biomédica en Red-(CB21/13/00083), Instituto de Salud Carlos III, Ministerio de Ciencia e Innovación and Unión Europea-NextGenerationEU. MC-J was the recipient of a CONTRATOS PREDOCTORALES DE FORMACIÓN EN INVESTIGACIÓN EN SALUD (PFIS) (FI22/00180) from the Instituto de Salud Carlos III (ISCIII) and co-funded by the European Union. DC-M was the recipient of a Rio-Hortega (CM22/00176) grant from the Instituto de Salud Carlos III (ISCIII) and co-funded by the European Union. PL-L was the recipient of a Margarita Salas contract funded by Plan de Recuperación, Transformación y Resiliencia, NextGeneration EU.

## Conflict of interest

The authors declare that the research was conducted in the absence of any commercial or financial relationships that could be construed as a potential conflict of interest.

## Publisher’s note

All claims expressed in this article are solely those of the authors and do not necessarily represent those of their affiliated organizations, or those of the publisher, the editors and the reviewers. Any product that may be evaluated in this article, or claim that may be made by its manufacturer, is not guaranteed or endorsed by the publisher.

## Abbreviations

HEV: *Paslahepevirus balayani*
WHO: World Health Organization
RNA: ribonucleic acid
PCR: polymerase chain reaction
95% CI: 95% confidence interval
AH: animal handler
PH: pig handlers
BU: butcher
